# Probiotic *Lactobacillus Paracasei* Expressing a Nucleic Acid-Hydrolyzing Minibody (3D8 Scfv) Enhances Probiotic Activities in Mice Intestine as Revealed by Metagenomic Analyses

**DOI:** 10.3390/genes9060276

**Published:** 2018-05-29

**Authors:** Seungchan Cho, Dongjun Kim, Yongjun Lee, Eui-Joon Kil, Mun-Ju Cho, Sung-June Byun, Won Kyong Cho, Sukchan Lee

**Affiliations:** 1 Department of Genetic Engineering, Sungkyunkwan University, 2066, Seobu-ro, Jangan-gu, Suwon 16419, Korea; seungchan1007@gmail.com (S.C.); rlaehdwns1535@gmail.com (D.K.); 88yjl11@naver.com (Y.L.); meitantei007@naver.com (E.-J.K.); munju2004@naver.com (M.-J.C.); 2 Animal Biotechnology Division, National Institute of Animal Science (NIAS), Rural Development Administration (RDA), 1500, Kongjwipatjwi-ro, Iseomyeon, Wanju 55365, Korea; pcs1778@korea.kr; 3 Research Institute of Agriculture and Life Sciences, College of Agriculture and Life Sciences, Seoul National University, 1, Gwanak-ro, Gwanak-gu, Seoul 08826, Korea

**Keywords:** *Lactobacillus paracasei*, 3D8 scFv, metagenomics, mouse, probiotics, 16S ribosomal RNA gene

## Abstract

Probiotics are well known for their beneficial effects for animals, including humans and livestock. Here, we tested the probiotic activity of *Lactobacillus paracasei* expressing 3D8 scFv, a nucleic acid-hydrolyzing mini-antibody, in mice intestine. A total of 18 fecal samples derived from three different conditions at two different time points were subjected to high-throughput 16S ribosomal RNA (rRNA) metagenomic analyses. Bioinformatic analyses identified an average of 290 operational taxonomic units. After administration of *L. paracasei*, populations of the probiotics *L. paracasei*, *Lactobacillus reuteri*, and *Pediococcus acidilactici* increased, whereas the population of harmful bacteria such as *Helicobacter* species decreased. Furthermore, continuous administration of *L. paracasei* resulted in *L. paracasei* emerging as the dominant probiotic after competition with other existing probiotics. Expression of 3D8 scFv protein specifically increased the population of *P. acidilactici*, which is another probiotic. In summary, our results showed that *L. paracasei* expressing 3D8 scFv protein enhanced probiotic activity in mice intestine with no observable side effects. Thus, the system developed in this study may be a good tool for the expression of recombinant protein using probiotics.

## 1. Introduction

The microbiota in the gastrointestinal (GI) tracts of mammals are complex, and their composition can be influenced by several factors, including diets and environmental changes. Among known microbiota in the GI tracts, some bacteria species are regarded as probiotics, which can be defined as microorganisms providing beneficial effects when consumed by humans and animals [1]. Of known probiotics, many members of *Lactobacillus* spp. are consumed as health supplementary foods [2] and are used in the food fermentation industry to ferment cereals inhibiting pathogenic bacteria [3]. *Lactobacillus* are gram-positive bacteria with a rod-like shape that participate in glucose fermentation resulting in the production of lactic acid as well as small amounts of acetic and succinic acids [4]. *Lactobacillus* usually inhabits the mouth and GI tract [5]. In particular, the GI tracts of diverse mammals are preferentially colonized by *Lactobacillus* spp. such as *Lactobacillus brevis*, *Lactobacillus casei*, *Lactobacillus acidophilus*, *Lactobacillus plantarum*, *Lactobacillus fermentum*, and *Lactobacillus salivariu* [6]. To date, more than 70 different species of *Lactobacillus* have been identified. 

The 3D8 single-chain variable fragment referred as 3D8 scFv is a nucleic acid hydrolyzing mini-antibody without sequence specificity [7]. The 3D8 scFv protein that was purified from *Escherichia coli* was subsequently shown to penetrate the cytosol of HeLa cells via caveolae-mediated endocytosis. Previous studies demonstrated that 3D8 scFv exhibits antiviral effects against a broad range of viruses, including the herpes simplex virus (HSV), pseudorabies virus (PRV), classical swine fever virus (CSFV), murine norovirus (MNV) and H1N1 influenza virus in various hosts [8,9,10,11]. Therefore, it is clear that 3D8 scFv engages in direct antiviral activities against various DNA and RNA viruses by penetrating into cells and directly hydrolyzing the viral genome based on previous findings. In addition, a previous study has shown that *Lactobacillus paracasei* expressing 3D8 scFV can be used as a preventive probiotic against norovirus infection [8]. In order to use *L. paracasei* expressing 3D8 scFV as a preventive probiotic, it is necessary to examine the possible impact of *L. paracasei* expressing 3D8 scFV in microbiota of the GI tracts. Because nothing is known about whether *L. paracasei* expressing 3D8 scFV will be beneficial or harmful to other microbiota. For this reason, metagenomics might be a good approach. 

The rapid development of next-generation sequencing (NGS) has promoted studies associated with metagenomics, which reveals the composition of living microorganisms in a wide range of environmental samples [12]. In the case of bacteria, amplification of a partial 16S ribosomal RNA (rRNA) gene sequence followed by NGS is widely used as a culture-independent microbiological method. NGS-based 16S rRNA target sequencing allows identification of numerous bacteria species present in the intestine of animals. For example, it is estimated that there are approximately 40,000 bacterial species in the gastrointestinal microbiome of humans [13], and a recent study generated a reference mouse gut metagenome derived from fecal samples of 184 mice with different genetic backgrounds revealing 541 bacteria species [14]. Of known NGS systems, many previous studies used the FLX 454 system (454 Life Sciences, Branford, Connecticut, US), which produces long length sequence reads [15], whereas recent studies preferentially adopted the MiSeq system (Illumina, San Diego, California, US), which can produce reads up to 300 bases in length for 16S rRNA target sequencing [16]. 

In this study, we carried out high-throughput 16S rRNA metagenomic analyses to investigate the effects of the probiotic *L. paracasei* ATCC334 expressing 3D8 scFv in microbiota of the mouse intestine. A total of 18 fecal samples derived from three different conditions at two different time points were subjected to high-throughput 16S rRNA metagenomic analyses. Our results showed that *L. paracasei* expressing 3D8 scFv promoted probiotic activity in the mouse intestine.

## 2. Methods

### 2.1. Animals and Bacteria 

Six-week-old female specific pathogen-free (SPF) BALB/c mice (Orient Bio Laboratories, Seongnam, Korea) weighing 18–20 g were housed under standard laboratory conditions. All animal procedures performed in this study were reviewed, approved, and supervised by the Institutional Animal Care and Use Committee (IACUC) of Kunkuk University (Ethical code: KU16080) and all experimental procedures performed here were in accordance with the guidelines of the Institute of Laboratory Animal Resources (ILAR). *L. paracasei* ATCC 334 was kindly provided by Dr. Jos Seegers (Falcobio, Lieden, Netherland). Wildtype (WT) *L. paracasei* and transgenic (TG) *L. paracasei* were anaerobically cultured in antibiotic-free de Man, Rogosa, and Sharpe (MRS) media and MRS media supplemented with 3 μg/mL chloramphenicol, respectively. 

### 2.2. Construction of a Vector Expressing Recombinant Protein and Transformation

A pSLP111.3 vector for expression in *Lactobacillus* (provided by Dr. Jos Seegers) was modified to replace the xylose-inducible promoter with a lactate dehydrogenase (LDH) constitutive promoter [17]. Codon-optimized 3D8 scFv was chemically synthesized (IDT, Coralville, IA, USA). Cloning of the 3D8 scFv gene into *L. paracasei* was performed as previously described [8]. 

### 2.3. Oral Administration of *Lactobacillus* to BALB/c mice

**Figure 1:**
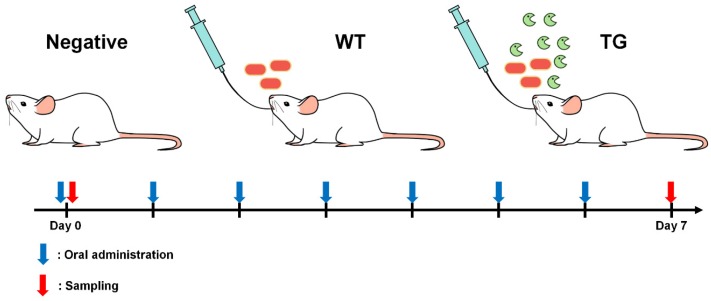
Experimental scheme of sample preparation for metagenomic study. Three different conditions were used: negative, WT, and TG. Syringes indicate normal oral administration. Orange rods and green shapes indicate *Lactobacillus* and *Lactobacillus* expressing 3D8 scFv, respectively. Blue arrows indicate time points for oral administration while red arrows indicate sampling time points of day 0 and day 7. WT: Wildtype; TG: Transgenic

Mice were assigned to three experimental groups, with three mice per group. The administration scheme is illustrated in Figure 1. For the three experimental groups, 6-week-old female BALB/c mice were fed 10^8^ colony forming units (CFU) of WT and TG *L. paracasei* once a day for 7 days using oral zonde needle. Mice in the negative control group were fed only with PBS once a day for 7 days.

### 2.4. Sample Preparation and DNA Extraction

Fecal samples were collected from the mice 7 days after administration. Genomic DNA was extracted from a total of 18 samples, including three biological replicates for each condition, using ExtractMaster™ Fecal DNA Extraction Kit according to the manufacturer’s instructions (Epicentre, Madison, WI, USA). 

### 2.5. Preparation of Libraries for 16S Ribosomal RNA Sequencing and Paired-End Sequencing Using the MiSeq System

Libraries were constructed following the Illumina 16S metagenomics sequencing library preparation guide. In brief, the V3 and V4 regions of the 16S rRNA gene were targeted for PCR amplification using isolated DNA as a template. The 7-bp barcoded PCR primers were used to label 16S rRNA amplicons for each individual sample. Paired-end sequencing (2 × 300 bp) was conducted using the MiSeq system (Illumina Inc., San Diego, CA, USA) at Macrogen (Seoul, Korea). The raw images generated by Illumina MiSeq system were processed using MiSeq Control Software (MCS, v2.2, Illumina Inc.) for system control with base calling by integrated primary analysis software called Real Time Analysis (RTA, v1.18, Illumina Inc.). The binary base calls (BCL) is converted into FASTQ using the Illumina package MiSeq Reporter (MSR). The obtained raw data were deposited in Sequence Read Archive (SRA) database in National Center for Biotechnology Information NCBI with project number PRJNA352304. 

### 2.6. Preprocessing of Raw Data, Clustering, and Taxonomic Assignment 

Data analyses consisted of three steps: preprocessing and clustering, taxonomic assignment, and statistical analyses for diversity. Paired-end reads were merged using the FLASH program [18]. The filtered clean reads were subjected to clustering at 100% identity to identify operational taxonomic units (OTUs) using the CD-HIT-OUT program [19]. Clustering was comprised of three steps. First, short reads and extra-long tails were removed from the raw data. The second step was to identify error-free reads. In the third step, the remaining representative reads from non-chimeric clusters were clustered using a greedy algorithm into OTUs with 97% identity at species level. Taxonomical composition from phylum to species level for each sample was analyzed using the quantitative insight into microbial ecology (QIIME) pipeline [20]. Community richness and diversity (α diversity) were analyzed by diverse α diversity metrics: Chao1, Shannon, Simpson, and Good’s coverage using the QIIME pipeline. 

### 2.7. Statistical Analyses

We have conducted a power test using the G*Power program (version 3.1.9.2) to calculate the power of the study [21]. Post hoc testing to compute the achieved power was conducted by one-way analysis of variance (ANOVA) using an *F*-test with the following parameters: Repeated measures, within-between interaction, effect size *f* 0.4 (large), α error probability 0.05, number of groups 3, number of measurements 2, correlation among replicate measures 0.05, nonsphericity correction 1, resulting in a power of 0.7882055 (78.82). In order to achieve a power higher than 0.8 (80) with the same parameters, the sample size should be more than 42, with an expected power of 0.8034136. 

In addition, a *t*-test was conducted on five major species of *Helicobacter* sp., *Pediococcus acidilactici*, *L. paracasei*, *L. reuteri*, and *Bifidobacterium* using the following parameters: difference between two dependent means (matched pairs), correlation between groups 0.5, α error probability 0.05, total sample size 6, and respective effect size. Effect sizes for two different groups was determined from group parameters (mean value group 1, mean value group 2, standard deviation (SD) value group 1, SD value group 2) using the G*Power program. 

Next, we conducted a two-way ANOVA with replication using Excel's Data Analysis (version 2016, Microsoft, Redmond, WA, USA) to find bacteria species significantly changed under three different conditions (negative (N), TG and WT) and two time points. 

The differences in abundance of bacterial genera among 18 samples, the ANOVA was performed using the Statistical Analysis of Metagenomic Profiles (STAMP v. 2.1.3) software package [22]. For comparison between two different conditions, two-sided Fisher’s exact test with confidence interval (CI) method (DP: Asymptotic-CC with 0.95) was conducted using STAMP software package. 

## 3. Results

### 3.1. Sample Preparation and High-Throughput 16S ribosomal RNA Metagenomic Analyses Using MiSeq System

Negative samples were obtained from the mice fed with PBS buffer or fed a diet containing WT samples were from mice fed *L. paracasei*, and TG samples were from mice fed *L.paracasei* expressing 3D8 scFv, as established in a previous study [8]. To examine changes in the microbiota in mice intestines among different conditions, we harvested fecal samples at two different time points (day 0 and day 7) for each condition (Figure 1 and Table 1). Finally, a total of 18 samples derived from three different conditions at two different time points with three biological replicates were subjected to metagenome profiling. A total of 5,089,513 reads (2,302,478,088 bp) were obtained from 18 libraries (Table 1). Between 224,927 reads (102,570,095 bp) and 326,110 reads (148,592,201 bp) were obtained from each sample (Table 1). The power of our study was 0.7882055 (78.82) with three number of groups (three conditions), two number of measurements (two time points), and three replicates for each group.

### 3.2. Microbial Composition in Mice Intestine

**Figure 2:**
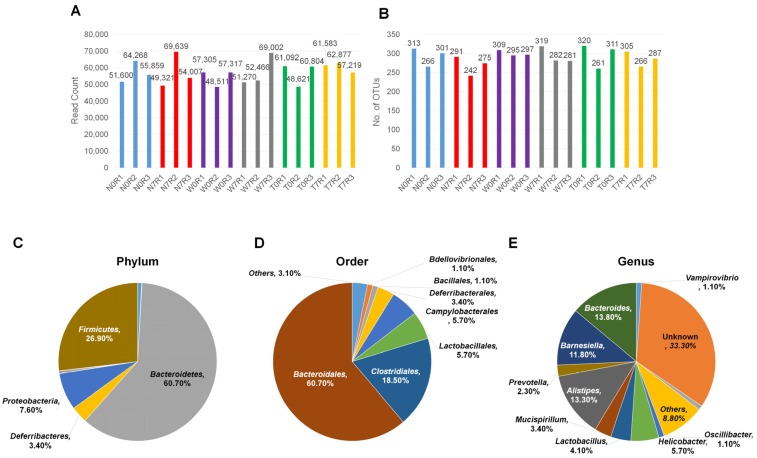
Analyses of sequenced reads in each library and assignment of all filtered reads according to bacterial taxonomy. (**A**) Number of analyzed reads in each library, (**B**) number of identified operational taxonomic units (OTUs) in each library, (**C**–**E**) taxonomical classification of all analyzed reads from 18 libraries according to phylum (**C**), order (**D**), and genus (**E**).

We carried out bioinformatics analyses for NGS data as described in methods section. Because of the stringent criteria for filtering sequenced reads, approximately 21% to 27% of sequenced raw data were further used for the identification of OTUs. We obtained from 48,511 reads (W0R2) to 69,639 reads (N7R2), which was enough for OTU analyses (Figure 2A). There was some bias for the number of filtered reads among replicates. In total, we identified 396 OTUs from 18 libraries (Table S1). In detail, between 242 OTUs (N7R2) and 320 OTUs (T0R1) were identified, with an average of 290 OTUs (Figure 2B). Based on our results, there was no correlation between number of reads and number of OTUs.

To reveal microbial composition in the mice intestine, we combined all data and classified the microbes into phylum, order and genus. We identified 9 phyla, 13 classes, 16 orders, 31 families, and 52 genera. Based on phylum, *Bacteroidetes* (60.70%) was dominant followed by *Firmicutes* (26.90%), *Proteobacteria* (7.60%), and *Deferribacteres* (3.40%) (Figure 2C). According to the order, most bacteria belonged to the *Bacteroidales* (60.70%) followed by *Clostridiales* (18.50%), *Lactobacillales* (5.70%), *Campylobacterales* (5.70%), and *Deferribacterales* (3.40%) (Figure 2D). However, many bacteria (33.30%) were not assigned into any known bacteria genera. The *Lactobacillus* accounted for 4.10% of all identified bacteria in the mice intestine. 

**Figure 3:**
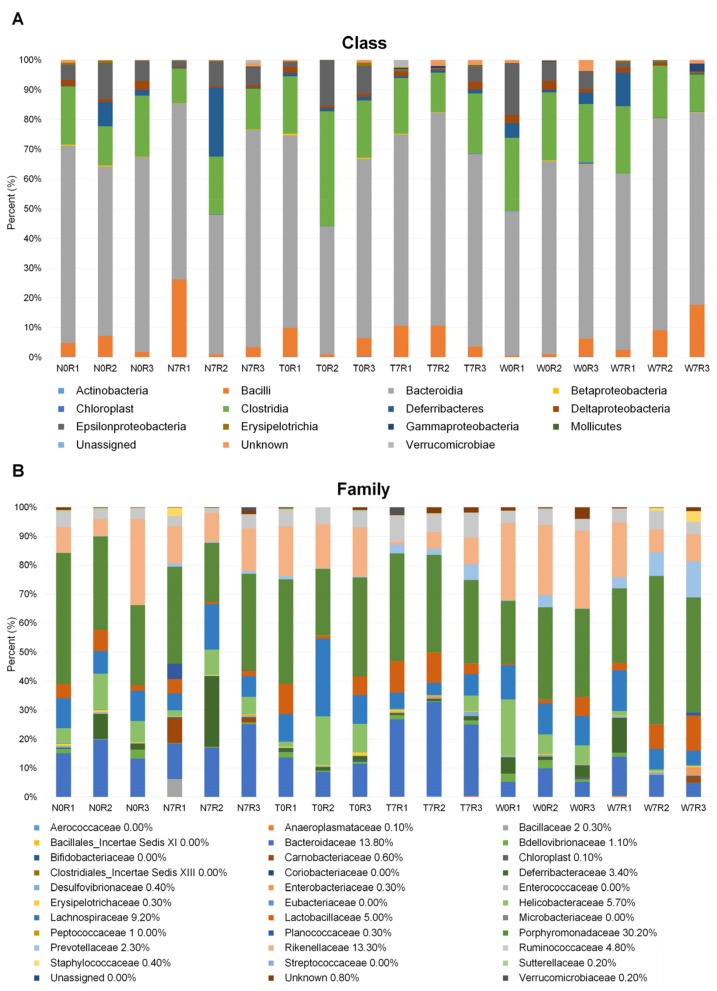
Taxonomical classification of OTUs in 18 samples. The identified OTUs were classified according to class (**A**) and family (**B**). Each color bar indicates the relative proportion of identified bacteria.

Among 13 identified known classes, the proportion of bacteria belonging to an unknown class ranged from 0.5% (W0R1) to 26.10% (N7R1) (Figure 3A). The proportion of the class *Bacteroidia* ranged from 42.6% (T0R2) to 72.7% (N7R3). The second dominant bacterial class was *Clostridia* ranging from 11.5% (N7R1) to 38.2% (T0R2). The class *Epsilonproteobacteria* was also abundantly present in all samples. The class *Deferribacteres* was identified in most samples except W7R2, and compared with other samples, the proportion of class *Deferribacteres* was very high in the N7R2 sample. 

**Figure 4:**
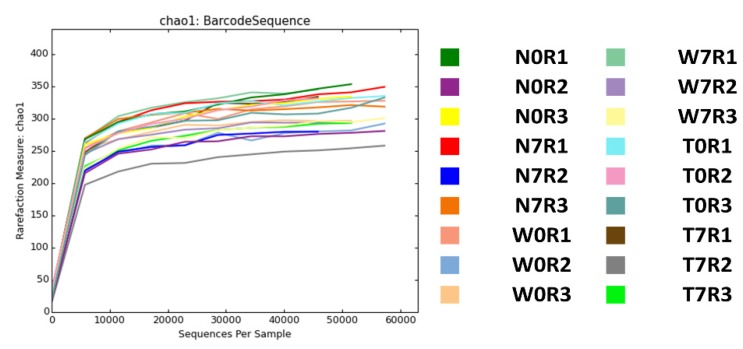
Rarefaction curves for 18 samples based on the OTUs.

Among the 31 known bacterial families, only 0.8% of bacteria were identified as unknown (Figure 3B). The three bacterial families *Porphyromonadaceae*, *Bacteroidaceae*, and *Rikenellaceae* were abundantly present in all samples. Rarefaction curves based on the Chao1 index were generated for 18 libraries and showed that the rarefaction curves nearly reached a plateau (Figure 4). Sequencing coverage for the 18 samples ranged from 0.9991079 to 0.999704 (Table 2). Both rarefaction and sequencing coverage indicate sufficient coverage of 16S rRNA sequencing. In addition, we calculated the richness estimate and diversity index using the Mothur program [23]. Although there was significant bias for the richness estimate and diversity index among biological replicates in the same condition, we did not find any significant differences among conditions (Table 2).

### 3.3. Comparison of Bacterial Compositions Among the Three Different Conditions

**Figure 5:**
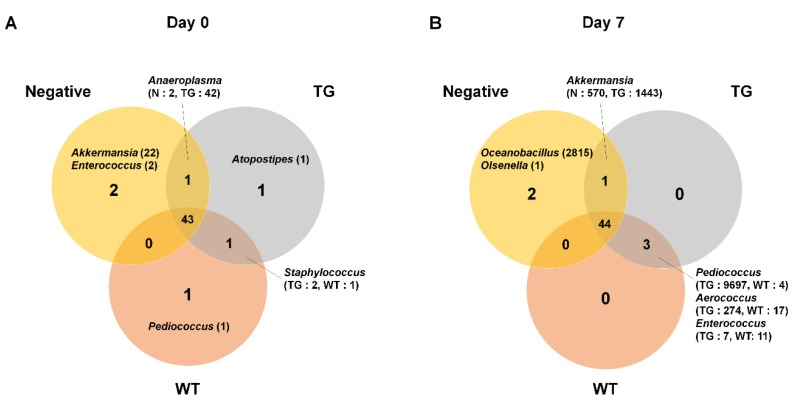
Comparison of identified bacteria in three different conditions based on genus. The identified bacterial genera in each condition were compared at two individual time points of day 0 (**A**) and day 7 (**B**) and visualized by Venn-diagram. Orange, gray, and pale red circles indicate N, TG, and WT conditions, respectively. Bacteria genera that were condition specific or commonly identified in two conditions were indicated with number of read counts. For each condition, bacterial genera from three replicates were combined regardless of number of read counts.

To identify condition-specific bacterial genera, we analyzed the composition of bacterial genera in three conditions at two different time points (Figure 5). A total of 49 and 50 genera were identified at day 0 and day 7, respectively. Most of the identified bacterial genera were commonly identified in all three conditions. For example, 43 genera (87.8%) and 44 genera (88%) were commonly identified at day 0 and day 7, respectively. At day 0, the two genera *Akkermansia* and *Enterococcus* were specific for the negative condition whereas *Atopostipes* and *Pediococcus* were specific for TG and WT, respectively. The read counts for condition specific bacterial genera were very low, ranging from only 1 count to 22 counts. At day 7, the genera *Oceanobacillus* and *Olsenella* were specific for negative condition. Interestingly, the read count for the genus *Oceanobacillus* was very high (2815 reads). The genus *Akkermansia* was commonly identified in negative and TG conditions relatively high read counts (N: 570, TG: 1443). Furthermore, between TG and WT conditions, the three genera *Pediococcus* (TG: 9697, WT: 4), *Aerococcus* (TG: 274, WT: 17), and *Enterococcus* (TG: 7, WT: 11) were identified. In particular, the number of reads associated with the genus *Pediococcus* was very high in the TG condition. 

**Figure 6:**
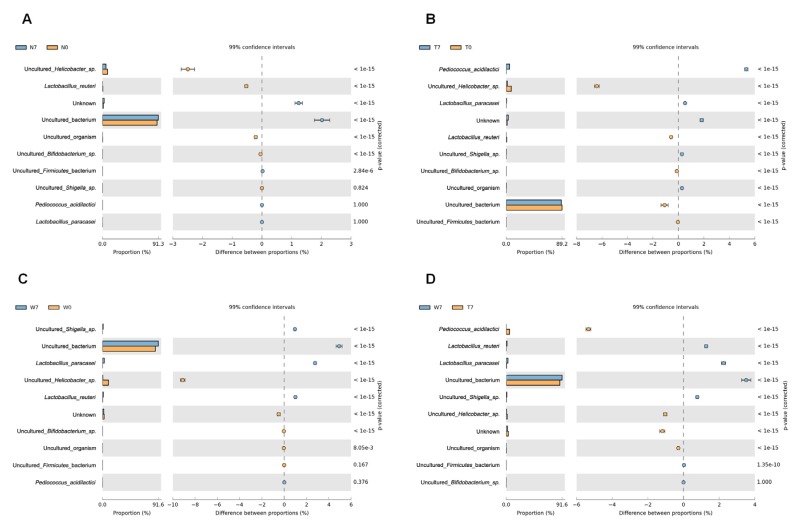
Comparison of bacterial species between two different groups. The difference in abundance of bacterial species between two different groups such as (**A**) N0 vs N7, (**B**) T0 vs T7, (**C**) W0 vs W7, and (**D**) T7 vs W7 were visualized by extended error bar plots implemented in STAMP program. The mean portions (%) and difference between portions were indicated with corrected *p* values.

We further analyzed the identified bacteria according to species in order to decipher changes in bacterial composition among the three different conditions (Table 3). In total, 1,032,761 reads were identified from 18 samples. Of these, 89.37% of reads were derived from uncultured bacteria, followed by uncultured *Helicobacter* sp. (5.63%), and unknown species (2.35%). At the species level, there were dramatic changes in microbial composition among conditions (Figure 6). *L. paracasei* that was used for feeding was not found in N0, N7, T0, or W0, as expected (Table 3 and Figure 6A–C). At 7 days after feeding, a large number of *L. paracasei* were found in both TG and WT samples (Figure 6B,C). The number of *L. paracasei* in WT at day 7 (4,790 reads) was about five times larger than that in TG at day 7 (953 reads). Interestingly, we also identified another lactobacillus, *L. reuteri* that was not fed to the mice. The amount of *L. reuteri* was decreased in negative and transgenic samples at day 7 compared day 0, whereas the amount of *L. reuteri* was increased in wildtype at day 7 (Figure 6B,C). In addition, we identified *P. acidilactici*, which is a known probiotic. Surprisingly, the population of *P. acidilactici* was dramatically increased in the transgenic condition at day 7 (Figure 6D). However, the number of uncultured *Bifidobacterium* species was decreased in all three conditions at day 7 compared with day 0 (Figure 6A–C).

We identified not only probiotics, but also potential pathogenic bacteria such as uncultured *Firmicutes* bacterium, uncultured *Helicobacter* species, and uncultured *Shigella* species. Of these, the number of *Helicobacter* species was decreased in all conditions at day 7 compared with day 0 (Figure 6A–C). 

We calculated the power based on five major species of *Helicobacter* sp., *P. acidilactici*, *L. paracasei*, *L. reuteri*, and *Bifidobacterium*. As a result, we obtained a power greater than 0.8 following three bacteria species in a given comparison, such as a power of 0.8556975 (T0 vs. T7) and 0.8820874 (W0 vs. W7) for *Helicobacter* sp., a power of 0.9914611 (N7 vs. T7) and 0.9825466 (W0 vs. W7) for *P. acidilactici*, and a power of 0.8329246 (W0 vs. W7), 0.9139413 (T7 vs. W7), and 0.9370286 (W7 vs. N7) for *Lactobacillus reuteri*. Furthermore, a two-way ANOVA test demonstrated that the abundance of *Helicobacter* sp. (*p* = 0.020955727) was significantly changed among the three conditions, while *P. acidilactici* showed significant changes in abundance by time (*p* = 0.007410375), condition (*p* = 0.00245994), and by the interaction between time and condition (*p* = 0.002458016).

**Figure 7:**
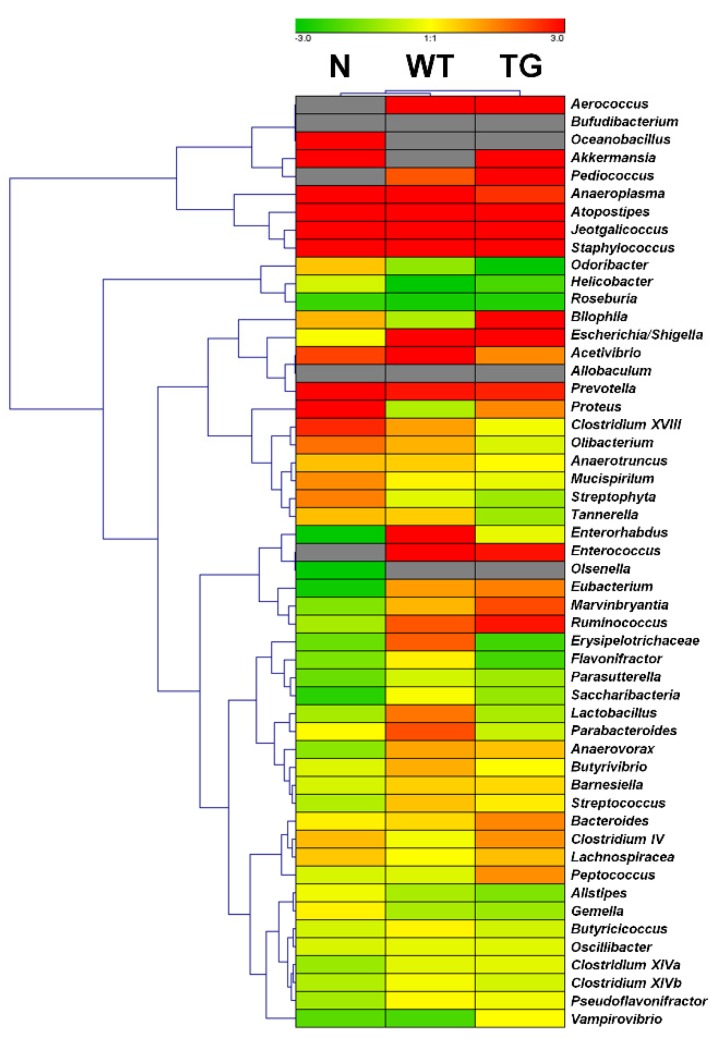
Relative abundance of known bacterial genera in three different conditions. Read numbers for 52 known bacteria genera in the same condition were combined. For each condition, the number of reads at day 7 was compared with the number of reads at day 0. The fold changes were converted into log_2_ and imported into the Genesis program. Hierarchical clustering was performed using the complete linkage method. Red and green colors indicate up- and down-regulation, respectively. A gray color indicates that the number of reads associated with the respective bacterium was 0.

We examined the relative abundance of the 52 known bacteria genera in three conditions at two different time points. The relative abundance of selected bacteria at day 7 was compared to that at day 0. The heat map shows changes in bacteria abundance under the three conditions (Figure 7). Bacteria belonging to the genera *Aeroccus* (*p* = 0.237), *Pediococcus* (*p* = 0.093), and *Enterococcus* (*p* = 0.060) were highly present in WT and TG, but not in the N sample (Table S2). Genera *Oceanobacillus* (*p* = 0.332) and *Olsenella* (*p* = 0.006) were only present in N samples; however, *Oceanobacillus* (*p* = 0.332) was highly abundant at day 7, whereas *Olsenella* (*p* = 0.006) was highly abundant at day 0. The abundance of bacteria belonging to the genera *Anaeroplasma* (*p* = 0.067), *Atopostipes* (*p* = 0.167), *Jeotgalicoccus* (*p* = 0.176), *Staphylococcus* (*p* = 0.105), and *Prevotella* (*p* = 0.024) was high at day 7 in all three conditions.

## 4. Discussion

Mice are frequently used as a model animal to examine the changes in microbiota in various tissues in response to diverse environmental stimuli. Modulation of the microbiota under a given condition results in a beneficial or harmful effect on the host life. For instance, many studies have shown the effects of antibiotic treatment on the mouse intestinal microbiome [24,25,26]. In addition, some studies have demonstrated the influence of dietary ingredients on the fecal microbial community in mice [27,28]. Moreover, the change of mouse intestinal microbiota in response to different environmental changes such as irradiation [29], acclimatization-induced stress [30], and exposure to arsenic and iron [31] has been studied. Gut microbiota from mice with different genetic backgrounds such as wildtype mice and gnotobiotic mice have also been previously examined [32,33]. 

In this study, we performed metagenome sequencing of a mouse model to examine the impact of consumption of lactobacillus expressing a recombinant protein on mouse intestine microbiota. We identified approximately 300 species from a single source mouse which represents about 60% of known bacteria species in a reference mouse gut metagenome derived from 184 mice [14]. Based on this result, our metagenomic approach was successful for revealing the microbiota in the intestine of a single source mouse. Among three biological replicates used for each condition, the microbial compositions among different replicates for the same condition were similar, but the abundance of identified bacteria was diverse. These results indicate that each individual mouse has a unique complex of microbiota, as previously shown [14].

Of the known top core genera in the mouse gut metagenome, three genera, *Bacteroides*, *Alistipes*, and *Lactobacillus*, were identified in our study; however, we additionally identified *Barnesiella*, *Mucispirillum*, and *Oscillibacter* genera. Of these, bacteria in the genus *Bacteroides* are clinical pathogens present in most anaerobic infections and have a beneficial relationship with the host [34]. In addition, *Barnesiella* is an obligatory anaerobic bacteria belonging to the *Bacteroidetes* and is one of the bacteria most frequently identified in the gut microbiota of BALB/c mice, which were used in our study [35]. 

Numerous studies have demonstrated that many factors influence the composition of microbiota. Of factors known to influence the composition of microbiota, probiotics are the main living factors that positively affect host health. For example, a transcriptome-based study showed that *B. bifidum*, a probiotic, reduced the cholesterol level in mice [36]. Colonization of *B. bifidum* in the intestine of different mice was monitored by quantitative real-time PCR [37]. Other studies using lactobacillus species showed a reduction in obesity [38] and inhibition of alcohol-induced pathogenic activity in the liver [39]. In addition, several previous studies using metagenomic approaches showed that probiotics modulate the intestinal microbiota [40]. Furthermore, a recent study demonstrated that the probiotic *L. casei* expressing human lactoferrin confers antibacterial activity in the GI tract of mouse [41]. 

In this study, we found that the administration of lactobacillus resulted in an increased population of the introduced *L. paracasei*. However, the populations of other probiotics *L. reuteri* and uncultured *Bifidobacterium* spp., which were already present in the intestine of the mouse, were decreased. We suppose that this result reflects competition among different probiotics, with the introduced *L. paracasei* becoming the dominant probiotic as a result of its continuous administration. In contrast, the population of uncultured *Helicobacter* sp., which are harmful bacteria, was decreased by administration of lactobacillus. These results are highly consistent with the known ability of probiotics to increase populations of probiotics and decrease populations of harmful bacteria.

The heat map identified several bacteria genera whose abundance was dramatically increased by the administration of lactobacillus. In particular, several round-shaped bacteria such as the genera *Aerococcus*, *Pediococcus*, *Enterococcus*, and *Ruminococcus* were identified. In general, members of the genera *Pediococcus* and *Enterococcus* are lactic acid bacteria of the phylum *Firmicutes* [42]. Therefore, we cautiously presume that the identified bacteria belonging to the genera *Aerococcus* and *Ruminococcus* might be similar to other lactic acid bacteria. 

We also identified a large number of uncultured bacteria that are unknown. Although these included several bacteria species that might be involved in probiotic activity, most of them were unknown bacteria for which genome and basic characteristics are not currently available. Therefore, identification and characterization of unknown bacteria residing in the mouse intestine should be intensively conducted.

The most interesting result was a dramatic increase in the population of *P. acidilactici*, which is a probiotic in the T7 condition. The number of reads for *P. acidilactici* was one in W0 and four in W7 conditions. This result indicates the presence of *P. acidilactici* in the BALB/c mouse. Interestingly, administration of *L. paracasei* expressing 3D8 scFv facilitated growth by *P. acidilactici*, whereas administration of *L. paracasei* without 3D8 scFv did not change the population of *P. acidilactici*. Based on this result, we cautiously propose a positive relationship between expressions of 3D8 scFv and *P. acidilactici*. 

We hypothesized that the newly developed system composed of lactobacillus expressing 3D8 scFv would not affect the microbial composition in the mouse intestine. However, the influence of lactobacillus expressing 3D8 scFv was very species specific. It might be of interest to further examine the correlation between expression of 3D8 scFv and *P. acidilactici*. Furthermore, it would also be interesting to test other recombinant proteins for the ability to change the microbiota in the intestine of mice. We believe that the newly established system expressing a recombinant protein might be a useful tool for probiotics-related research or industry.

Taken together, our results showed that probiotic *L. paracasei* expressing 3D8 scFv protein enhances probiotic activity in the mice intestine without any observable side effects. Thus, the system developed in this study might be a good tool for expression of recombinant protein using probiotics in the near future.

## Figures and Tables

**Table 1:** Summary of treatment conditions and samples with respective sequencing results.

Condition	Day	Replicate	Abbreviation	Total Bases (bp)	Read Count
N	0	1	N0R1	117,395,034	259,801
		2	N0R2	129,075,064	285,466
		3	N0R3	128,072,181	283,889
	7	1	N7R1	102,570,095	224,927
		2	N7R2	147,077,671	325,948
		3	N7R3	113,530,929	250,331
WT	0	1	W0R1	129,201,892	288,118
		2	W0R2	125,601,235	278,505
		3	W0R3	140,795,712	311,553
	7	1	W7R1	121,644,286	269,049
		2	W7R2	123,340,544	271,652
		3	W7R3	148,592,201	326,110
TG	0	1	T0R1	138,288,303	305,165
		2	T0R2	107,080,820	237,986
		3	T0R3	135,852,937	300,959
	7	1	T7R1	139,324,032	307,834
		2	T7R2	131,123,910	287,958
		3	T7R3	123,911,242	274,262

Samples were harvested from three conditions at two different time points with three biological replicates for each. Group name, sampling time, name of replicate, and abbreviated name for individual sample are provided. In addition, total sequenced nucleotide bases and read counts for each sample by NGS are provided. N: negative; WT: wildtype; TG: Transgenic.

**Table 2:** Richness estimate and diversity index for gut samples under different diets: community diversity index and richness estimate for 18 samples.

Sample	OTUs	Chao1	Shannon	Simpson	Goods Coverage
N0R1	313	352.667	5.8142597	0.9593455	0.999321705
N0R2	266	282.235	5.0649192	0.9344073	0.999626564
N0R3	301	332.316	5.5026827	0.9550186	0.999373422
N7R1	291	338.3	5.6458194	0.9655083	0.999107885
N7R2	242	264.235	4.4974754	0.9035892	0.999597926
N7R3	275	295.313	5.4140089	0.9521503	0.999518581
W0R1	309	335.4	5.4151359	0.9425895	0.999424134
W0R2	295	334	5.8569545	0.9670265	0.999175445
W0R3	297	332.769	5.6102921	0.955642	0.999459148
W7R1	319	338.833	5.6387481	0.9569166	0.99931734
W7R2	282	295.043	5.610285	0.9572774	0.999523501
W7R3	281	307.105	5.6519495	0.9641348	0.999536245
T0R1	320	349	5.8469024	0.963316	0.999508937
T0R2	261	282.368	4.9104504	0.9228563	0.99940355
T0R3	311	320	5.810696	0.9640873	0.999703967
T7R1	305	330.588	5.631011	0.9554437	0.999512853
T7R2	266	295.077	5.140159	0.9231	0.999554686
T7R3	287	297.5	5.7108717	0.9602213	0.999632989

OTUs: Operational taxonomic unit is an operational definition of a species or group of species that is often used when only DNA sequence data are available; Chao1: The Chao calculator returns the Chao1 richness estimate for an OTU definition; Shannon: The Shannon index takes into account the number and evenness of species; Simpson: The Simpson index represents the probability that two randomly selected individuals in the habitat will belong to the same species; Goods Coverage: Goods Coverage calculated as C = 1 − (s/n), where s is the number of unique OTUs and n is the number of individuals in the sample. This index gives a relative measure of how well the sample represents the larger environment.

**Table 3:** Number of sequenced reads for known bacteria species in six conditions.

	All *	N0	N7	W0	W7	T0	T7
*Lactobacillus paracasei*	5743 (0.6%)	0	0	0	4790 (2.8%)	0	953 (0.5%)
*Lactobacillus reuteri*	6120 (0.6%)	1068 (0.6%)	163 (0.1%)	733 (0.4%)	2518 (1.5%)	1295 (0.8%)	343 (0.2%)
*Pediococcus acidilactici*	9702 (0.9%)	0	0	1	4	0	9697 (5.3%)
Uncultured bacterium	922,984 (89.4%)	153,395 (89.3%)	158,000 (91.3%)	141,368 (86.7%)	158,205 (91.6%)	152,017 (89.2%)	159,999 (88.1%)
Uncultured *Bifidobacterium* sp.	360	82	0	53	0	225 (0.1%)	0
Uncultured *Firmicutes* bacterium	275	14	52	65	53	84	7
Uncultured *Helicobacter* sp.	58,145 (5.6%)	14,060 (8.2%)	9849 (5.7%)	16,001 (9.8%)	1213 (0.7)	13,879 (8.1%)	3143 (1.7%)
Uncultured organism	2469 (0.2%)	594 (0.3%)	236 (0.1%)	307 (0.2%)	260 (0.2%)	274 (0.2%)	798 (0.4%)
Uncultured *Shigella* sp.	2669 (0.3%)	41	39	168 (0.1%)	1843 (1.1%)	40	538 (0.3%)
Unknown	24,294 (2.4%)	2473 (1.4%)	4628 (2.7%)	4437 (2.7%)	3852 (2.2%)	2703 (1.6%)	6201 (3.4%)
Total	1,032,761	171,727	172,967	163,133	172,738	170,517	181,679

At the species level, most identified bacteria were assigned as uncultured bacteria. Only a few bacteria species have been identified. Number of reads and percentage of respective bacteria species are indicated. * Six different conditions were used: Negative in Day 0 (N0), Negative in Day 7 (N7), WT in Day 0 (W0), WT in Day 7 (W7), TG in Day 0 (T0) and TG in Day 7 (T7).

## Data Availability

The obtained raw data were deposited in Sequence Read Archive (SRA) database in NCBI with project number PRJNA352304.

## Supplementary Materials

The following are available online at http://www.mdpi.com/2073-4425/9/6/276/s1, Table S1: Classification of identified 396 OTUs identified from 18 samples with respective bacteria taxonomy and read number., Table S2: Statistical analyses of 53 bacterial genera in 18 different samples by ANOVA implemented in STAMP program., Table S3: Read number, mean value, and standard deviation for the identified 396 species in each condition.
