# Supplementary material for: Probiotic Lactobacillus Paracasei Expressing a Nucleic Acid-Hydrolyzing Minibody (3D8 Scfv) Enhances Probiotic Activities in Mice Intestine as Revealed by Metagenomic Analyses
Source: Genes (Basel). 2018 May 29;9(6):276. doi: 10.3390/genes9060276 (PMC6027128; doi:10.3390/genes9060276)
Supplement: Supplementary file 1 [file genes-09-00276-s001.zip › Supplementary Information.docx]

**Supplementary Information**

**Probiotic *Lactobacillus paracasei* expressing a nucleic acid-hydrolyzing minibody (3D8 scFv) enhances probiotic activities in mice intestine revealed by metagenomic analyses**

Seungchan Cho^1^, Dongjun Kim^1^, Yongjun Lee^1^, Eui-Joon Kil^1^, Mun-Ju Cho^1^, Sung-June Byun^2^, Won Kyong Cho^3*^ and Sukchan Lee^1*^

^1^Department of Genetic Engineering, Sungkyunkwan University, 2066, Seobu-ro, Jangan-gu, Suwon 16419, Korea

^2^Animal Biotechnology Division, National Institute of Animal Science (NIAS), Rural Development Administration (RDA), 1500, Kongjwipatjwi-ro, Iseomyeon, Wanju 55365, Korea

^3^Department of Agricultural Biotechnology, College of Agriculture and Life Sciences, Seoul National University, 1, Gwanak-ro, Gwanak-gu, Seoul 08826, Korea

*Correspondence:
Dr. Sukchan Lee
[cell4u@skku.edu](mailto:cell4u@skku.edu)

Dr. Won Kyong Cho

[wonkyong@gmail.com](mailto:wonkyong@gmail.com)

**Supplementary data**

**Supplementary Tables**

**Table S1.** Classification of identified 396 OTUs identified from 18 samples with respective bacteria taxonomy and read number.

**Table S2.** Statistical analyses of 53 bacterial genera in 18 different samples by ANOVA implemented in STAMP program.

**Table S3.** Read number, mean value, and standard deviation for the identified 396 species in each condition.

Mean and SD indicate mean and standard deviation values, respectively. Green colored shells indicate five representative species, which were used for statistical tests. Gray colored shells indicate mean and SD values in each condition.
